# Incidence, Clinical Features, Associated Factors and Outcomes of Intensive Phase Antituberculosis Drug Induced Liver Injury Among Patients With Tuberculosis at a Tertiary Care Hospital in Nepal: A Descriptive Cross‐Sectional Study

**DOI:** 10.1002/hsr2.70686

**Published:** 2025-04-21

**Authors:** Ravi Ranjan Pradhan, Ajay Kumar Yadav

**Affiliations:** ^1^ Dhaulagiri Hospital Baglung Nepal; ^2^ National Academy of Health Sciences Kathmandu Nepal

**Keywords:** antitubercular therapy, drug induced liver injury, outcomes, risk factors, tuberculosis

## Abstract

**Background and Aims:**

Tuberculosis (TB) remains a significant global health concern, especially in Nepal, where the incidence of anti‐TB drug‐induced liver injury (DILI) is substantial. The main aim of this study is to investigate the incidence, clinical characteristics, outcomes, and contributing factors related to intensive phase anti‐TB DILI among TB patients.

**Methods:**

This prospective cross‐sectional study enrolled 78 TB patients. Patients received a weight‐based fixed‐dose Antitubercular therapy (ATT). Liver function tests (LFTs) were performed at baseline and periodically during treatment to monitor for anti‐TB DILI. Patients with DILI received immediate ATT discontinuation, supportive care, and reintroduction of ATT upon LFT normalization. Outcomes were tracked up to 60 days post‐DILI. Data were analyzed using SPSS v21. Statistical significance was set at *p* < 0.05.

**Results:**

The mean age of the patients was 49.87 years (SD = 18.61), and 57.7% were male. Anti‐TB DILI was observed in 15.4% of patients during the intensive treatment phase, with moderate severity in 50% of these cases. Half of the patients with DILI presented with nausea, vomiting, and anorexia. Notably, 91.7% of DILI patients showed improvement upon treatment discontinuation. The recurrence rate of anti‐TB DILI after ATT re‐initiation was 8.3%. Anti‐TB DILI developed at a median of 11 days (range: 7–60 days) after ATT initiation, with liver enzyme normalization after discontinuation of ATT averaging 10.9 ± 6.45 days. The mortality rate among DILI patients was 8.3% (1 out of 12 patients). Hepatotoxic drugs, low BMI, and low serum albumin were identified as independent predictors of anti‐TB DILI.

**Conclusion:**

Anti‐TB DILI occurred in a significant proportion of TB patients, with moderate severity being most common. Early detection and management, including treatment discontinuation, led to high recovery rates, though mortality remained notable. Low BMI, low serum albumin, and hepatotoxic drugs were key independent risk factors, emphasizing the need for careful monitoring and tailored management during ATT.

## 1 Introduction

Tuberculosis (TB) remains a significant public health concern in both developed and developing nations, primarily due to its rising prevalence among immunocompromised individuals, escalating medical costs, and the emergence of drug resistance [1, 2]. Short‐course antitubercular therapy (ATT) refers to a standard regimen used to treat TB, typically consisting of a combination of drugs: isoniazid (H), rifampicin (R), ethambutol (E), and pyrazinamide (Z), which are administered over a period of 6 months [3]. Nevertheless, one of the primary drawbacks of ATT is drug‐induced liver injury (DILI), which can lead to treatment interruption, noncompliance, treatment failure, and the development of drug resistance [4]. Globally, the incidence of DILI associated with anti‐TB treatment varies from 2.55% to 36.75% across different studies, depending on various definitions, study populations, and treatment protocols [3, 5, 6, 7, 8]. Mortality resulting from anti‐TB DILI ranges from 6% to 22.7% [9, 10], while it also contributes to increased morbidity, longer hospital stays, and elevated medical expenses. The precise mechanisms behind anti‐TB DILI remain unclear; however, several theories have been proposed, including direct toxicity of the compound or its metabolites, the generation of free radicals, immune‐mediated damage, and hypersensitivity reactions [11]. A study by Abbara et al. reported that 53% of anti‐TB DILI cases occurred within 2 weeks, and 87.6% within 8 weeks of initiating ATT [12]. Similarly, Tweed et al. found that liver enzyme monitoring during the first 2 months detected 75% of cases with peak enzyme elevation ≥ 3 × UNL [13].

Numerous studies have indicated that factors such as advanced age, female gender, chronic alcoholism, pre‐existing liver disease, hepatitis B virus carrier status, acetylator status, and nutritional status may predispose individuals to anti‐TB DILI [14, 15]. However, data from various countries lack consistency, making it challenging to pinpoint patients at a heightened risk of drug‐induced hepatitis.

There are two different methods of rechallenge of ATT: (a) *Regimen challenge*: Subjects are challenged with a combination of potentially hepatotoxic agents simultaneously, that is, HRZE. (b) *Step‐wise challenge*: Subjects challenged with one hepatotoxic agent at a time, separated by an interval of 3–8 days, until all drugs were reintroduced or hepatotoxicity recurred [16].

In Nepal, we frequently encounter cases of anti‐TB DILI in our daily clinical practice. Surprisingly, despite our experiences, only few prior studies have explored anti‐TB DILI in Nepal. Consequently, this study seeks to investigate the incidence, clinical characteristics, outcomes, and contributing factors related to intensive phase anti‐TB DILI among TB patients at a tertiary care hospital in Nepal. Furthermore, our study aims to investigate time of development of DILI after ATT initiation and the potential for DILI recurrence upon resuming ATT.

Our study findings will assist clinicians in predicting and managing anti‐TB DILI, ultimately helping reduce associated mortality. The results may also contribute to national health policy development for tuberculosis. Furthermore, the study aims to bridge gaps in existing knowledge on anti‐TB DILI in Nepal and could provide valuable insights for researchers considering large‐scale, multi‐center studies on this topic in the country.

## 2 Methods

### 2.1 Inclusion and Exclusion Criteria

**Figure 1:**
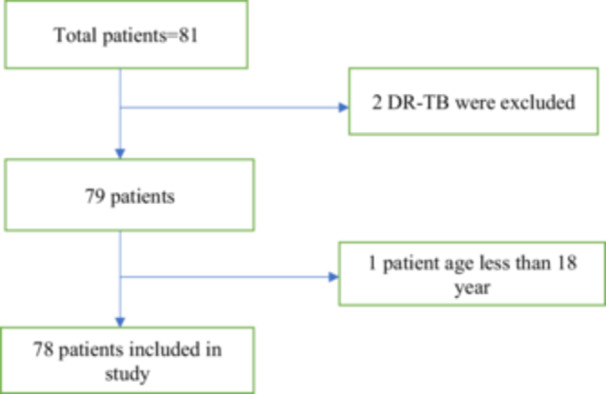
Flow chart showing patient selection based on inclusion and exclusion criteria.

This study included individuals aged 18 years and older who have been newly diagnosed with TB (either through bacteriological confirmation or clinical diagnosis), including carriers of HBsAg or chronic hepatitis C, with no known history of chronic liver disease or cirrhosis and with normal liver function tests (LFTs) or LFT values lower than twice the upper limit of normal (ULN) before initiating anti‐TB treatment; exclusion criteria encompass patients with confirmed chronic liver disease, LFTs values exceeding twice the ULN before treatment initiation, acute hepatitis (hepatitis A, B, C, E), a history of significant alcohol intake (more than two drinks per day for males and more than one drink per day for females), drug‐resistant TB, unwillingness to provide consent, and individuals under the age of 18 (Figure 1).

### 2.2 Study Design and Setting

This is a prospective cross‐sectional study conducted within a single healthcare institution over a duration of 12 months (August 2022 to August 2023). The study took place at the Madesh Institute of Health Sciences (MIHS), situated in the Madesh province of Nepal. MIHS is a tertiary care center with a capacity of 400 beds, serving as the primary healthcare facility in the proposed capital of the Madesh province. It offers a wide range of specialized medical services to patients hailing from all eight districts within the province. Patients who met the inclusion criteria were recruited from various departments, including the outpatient department, medicine ward, intensive care unit, and other hospital wards (Figure 1).

### 2.3 Study Methods

The diagnosis of TB was determined by the attending physician, relying on clinical, radiological, microbiological, biochemical, or histological findings.

Regarding the TB treatment regimen in Nepal, adults received a fixed‐dose regimen administered under Directly Observed Treatment Short‐course (DOTS) supervision. This regimen included Isoniazid, Rifampicin, Pyrazinamide, and Ethambutol (HRZE; 75 mg/150 mg/400 mg/275 mg) during the intensive phase and Isoniazid and Rifampicin (HR; 75 mg/150 mg) during the continuation phase. The dosage for adults was weight‐based, with patients weighing 20–30 kg receiving 1½ tablets, patients weighing 30–39 kg taking 2 tablets, patients weighing 40–54 kg taking 3 tablets, and patients weighing 55 kg and above taking 4 tablets of the fixed‐dose regimen.

All patients underwent a comprehensive medical history assessment, including screening for hepatitis B virus (HBsAg), hepatitis C virus (anti‐HCV), and HIV infections. For those who tested positive for HBsAg, additional tests such as IgM‐HBcAb, IgG‐HBcAb, HBeAg, and IgG‐HBeAb were conducted to confirm acute Hepatitis B virus infection. Patients testing IgM‐HBcAb negative, IgG‐HBcAb positive, HBeAg negative, and IgG‐HBeAb positive with normal LFTs were classified as HBsAg carriers. For individuals with positive anti‐HCV markers, the presence of HCV RNA was detected using polymerase chain reaction (PCR) to confirm acute hepatitis C virus infection. Patients with positive markers for acute hepatitis were excluded from the study.

Baseline LFTs, including AST, ALT, alkaline phosphatase, total bilirubin and its fractions, total proteins, and albumin, were conducted for all patients before starting ATT. The normal reference ranges were AST: 8–48 U/L, ALT: 7–55 U/L, alkaline phosphatase: 40–129 U/L, total bilirubin: 0.1–1.2 mg/dL, total proteins: 6.3–7.9 g/dL, and albumin: 3.5‐5 gm/dL. Subsequent LFTs were performed at the first and second weeks after initiating ATT, regardless of hepatitis symptoms [12, 13]. Patients were then followed up via phone contact at the fourth and eighth weeks to monitor for hepatitis symptoms (such as nausea, vomiting, jaundice, anorexia, and right upper quadrant pain), and repeat LFTs were performed in symptomatic patients [12, 13]. Patients were encouraged to report any hepatitis symptoms to their treating physician via phone contact or direct visit throughout the intensive phase of ATT, and LFTs were conducted in such cases.

The diagnosis of anti‐TB DILI adhered to the American Thoracic Society's definition, where ALT and AST levels must be at least three times the ULN when hepatitis symptoms are present or at least five times the ULN when no hepatitis symptoms are present. Patients were categorized into three groups based on the severity of DILI: mild (3–5 times UNL), moderate (5–10 times UNL), and severe (> 10 times UNL) [14, 17].

Patients diagnosed with DILI were admitted under the care of the attending physician. Immediate discontinuation of ATT was done, and standard supportive care for DILI was administered. The Model for End‐Stage Liver Disease (MELD) [18] score was calculated for each patient, and LFTs were repeated weekly until they normalize.

After LFTs returned to normal, ATT was reinitiated at a similar fixed‐dose regimen (i.e., regimen challenge) [16]. This regimen included Isoniazid, Rifampicin, Pyrazinamide, and Ethambutol (HRZE; 75/150/400/275 mg). Repeat LFTs were performed at the first and second weeks after rechallenge, regardless of hepatitis symptoms. Patients were monitored via phone contact at the fourth and eighth weeks for hepatitis symptoms, and LFTs were repeated in symptomatic patients [12, 13]. Patients were instructed to report any hepatitis symptoms to their treating physician, either through phone contact or through direct visit, during the entire intensive phase of ATT. Additionally, patient outcomes in terms of recovery, hepatic encephalopathy, chronic hepatitis, and death were monitored for up to 60 days after anti‐TB DILI.

*Sampling unit*: Patients with TB

*Sample size*:

$$n=z^{2}\text{pq}/L^{2},$$

where *n* is the required sample size; *z* the *Z*‐value corresponding to the desired confidence level (1.96 for 95% confidence); *p* the estimated prevalence of the condition (11%) [19]; *q* the complement of the prevalence, or 1−*p* (89%); *L* the allowable error, or the margin of error (7%). Using the above formula, the sample size was estimated to be 78.

### 2.4 Dropout Adjustment

Using the assumed dropout rate of 10% (or 0.10):

*n* (adjusted) = 78/(1 − 0.1), that is, 87.

So, the adjusted sample size was estimated to be 87.

#### 2.4.1 Explanation of the Parameters

2.4.1.1

*Prevalence*: The prevalence value of 11% (or 0.11) represents the estimated proportion of the population expected to have the condition, in this case, anti‐TB DILI. This value should ideally be based on local or regional epidemiological data. In our study, the prevalence was derived from a prior study conducted in India [19].

2.4.1.2

*Allowable error*: The allowable error, or margin of error, is set at 7% (or 0.07). This parameter reflects the degree of precision the study aims to achieve in estimating the population proportion. A 7% margin of error means that the true prevalence in the population is expected to lie within ±7% of the sample estimate, with 95% confidence.

2.4.1.3

*Justification for the adjusted sample size*: Incorporating this adjustment ensures that, even if 10% of participants are lost to follow‐up or do not comply with the study protocol, the study will still have 78 valid participants remaining, allowing for the calculation of prevalence with the same confidence level and margin of error.

### 2.5 Sampling Technique

Convenience non‐probability sampling.

### 2.6 Study Variables

*Dependent variables*: The dependent variables were the incidence and outcome of anti‐TB DILI (i.e., recovery, hepatic encephalopathy, chronic hepatitis and death).

*Independent variables*: The independent variables included socio‐demographic characteristics (age, gender), type of TB (extrapulmonary, pulmonary or disseminated), diagnosis modality (clinically diagnosed or bacteriological confirmed), severity of the TB, HBV, HCV and HIV infection status, CD4 count, use of other hepatotoxic drugs, and LFTs.

- *Pulmonary TB*: TB that primarily affects the lungs [20].
- *Extrapulmonary TB*: TB that affects organs outside of the lungs, such as the lymph nodes, pleura, peritoneum, pericardium, kidneys, bones, or meninges [20].
- *Disseminated TB*: Disseminated TB was diagnosed if TB involved two or more noncontiguous sites [20].
- *Bacteriological confirmed TB*: TB confirmed through laboratory tests, such as sputum smear microscopy, culture, or molecular techniques (e.g., PCR) [20].
- *Clinically diagnosed TB*: TB diagnosed based on clinical symptoms and signs (e.g., cough, fever, weight loss, imaging findings) without microbiological confirmation [20].
- *Severe TB*: Pericardial, military, meningeal, adrenal, and disseminated TB were categorized as severe TB [20].
- HBV infection: Identified through serological testing (HBsAg, HBV DNA levels).
- *HCV infection*: Identified through serological testing (anti‐HCV antibodies, HCV RNA testing).
- HIV infection: Identified through serological testing (HIV antibody or antigen tests, viral load).
- *Hepatotoxic drugs*: co‐trimoxazole, fluconazole, statins (e.g., atorvastatin), valporate, phenytoin, propylthiouracil, and herbal remedies were considered as hepatotoxic [5].

### 2.7 Tools and Techniques for Data Collection

*Data collection tools*: Structured interview.

*Data collection technique*: The process involved the use of an observation checklist crafted in English and customized to align with the study's objectives. This checklist encompassed socio‐demographic characteristics, disease‐related factors, patient attributes such as co‐morbidities, and outcome inquiries. Data collection was conducted by two carefully selected data collectors from the house officers of the internal medicine department, who underwent essential training to facilitate accurate data acquisition. The checklist's content validity was evaluated through expert assessment, ensuring that the meaning of each item was thoroughly scrutinized and adjusted as necessary.

*Consent*: The structured format of the interview ensured that informed consent was properly obtained and that participants were aware of the purpose and nature of the study. Patient's confidentiality was maintained. Sample of consent form is available at: https://doi.org/10.6084/m9.figshare.25975342.

*Ethical considerations*: Ethical clearance was obtained from institutional review committee (IRC) of Nepal health research council (NHRC) (Reg. No.: 321_2022).

*Validity and reliability of tools*: A pre‐test was administered to 10% of the sample size. Cronbach's alpha was computed for each domain, with a value of 0.7 or higher indicating sufficient internal consistency.

### 2.8 Statistical Analysis

We utilized EpiData v 3.1 for data entry and SPSS Statistics v 21 (IBM, Armonk, NY) for data analysis. The normality of data distribution was assessed using the Shapiro–Wilk test, revealing that age and serum albumin were not normally distributed, while BMI exhibited normal distribution. Continuous variables were presented as mean ± standard deviation (SD), while categorical variables were expressed as frequencies and percentages.

The association between anti‐TB DILI and age, as well as serum albumin, was examined using the Mann–Whitney *U* test as these variables were not normally distributed. The relationship between anti‐TB DILI and BMI was analyzed using an independent sample *t*‐test as the variable was normally distributed. The bivariate relationship between anti‐TB DILI and other risk factors was determined using chi‐square test as both the variables were categorical. Furthermore, multivariable logistic regression analysis was conducted to predict the independent risk factors for anti‐TB DILI. Statistical significance was set at a *p* value of < 0.05.

### 2.9 Potential Confounding Factors

Several potential confounding factors, such as alcohol use and co‐existing liver conditions, could affect the outcomes of the study. To address these potential confounders and ensure the validity of the study findings, the following considerations were made:

1. *Alcohol use:*
  The exclusion criterion for significant alcohol intake (defined as more than two drinks per day for males and more than one drink per day for females) aims to control for alcohol‐related liver damage, which could interfere with liver function and complicate the interpretation of liver‐related outcomes. By excluding individuals with heavy alcohol consumption, the study ensures that participants were not affected by alcohol‐induced liver injury, which could confound the assessment of liver function changes during ATT. It is important to note that the study did not account for moderate or occasional alcohol use, which may still contribute to liver damage, but the exclusion of individuals with significant alcohol use reduces the risk of this confounding factor.
2. *Co‐existing liver conditions:*
  To control for pre‐existing liver conditions, the study excluded individuals with chronic liver disease (such as cirrhosis or nonalcoholic fatty liver disease) and those with acute hepatitis (hepatitis A, B, C, or E). This ensures that the study population consists of individuals with relatively healthy liver function, thereby minimizing the risk of liver dysfunction arising from pre‐existing conditions that could confound the results. Furthermore, LFTs exceeding twice UNL were also excluded, ensuring that those with early signs of liver injury or hepatic impairment were not included, further isolating the effects of the ATT on liver function.
3. *HBV and HCV carriers:*
  The inclusion of individuals who are carriers of HBsAg or chronic hepatitis C adds complexity to the study, as these conditions could influence liver health. However, since participants were required to have normal LFTs or values below twice the UNL before starting ATT, the study attempts to control for any significant liver dysfunction at baseline.

## 3 Results

### 3.1 Demographic Characteristics of the Study Population

The study enrolled a total of 78 patients who met the inclusion criteria (Figure 1). The mean age was 49.87 years (SD = 18.61) and mean BMI was 21.74 (SD = 3.44) kg/m^2^. Out of the 78 patients, 45 (57.7%) were male and male to female ratio was 1.4:1. Diabetes (*n* = 11; 14%) and hypertension (*n* = 3; 3.8%) were the most common associated comorbidities (Table 1).

**Table 1:** Patients characteristics.

Parameters	Frequency	Percent
Gender		
Male	45	57.7
Female	33	42.3
Comorbidities		
None	64	82.1
Diabetes mellitus	11	14.1
Hypertension	3	3.8
Type of TB		
Pulmonary TB	48	61.5
Extra‐pulmonary TB	26	33.3
Disseminated TB	4	5.1
Modality of diagnosis		
Bacteriological confirmed	18	23
Clinically diagnosed	60	77
Severe TB		
Yes	6	7.7
No	72	92.3
HIV infection	0	0
HBV infection	0	0
HCV infection	0	0

Abbreviations: HBV, hepatitis B virus; HCV, hepatitis C virus; HIV, human immunodeficiency virus; TB, tuberculosis

### 3.2 Types of TB

**Figure 2:**
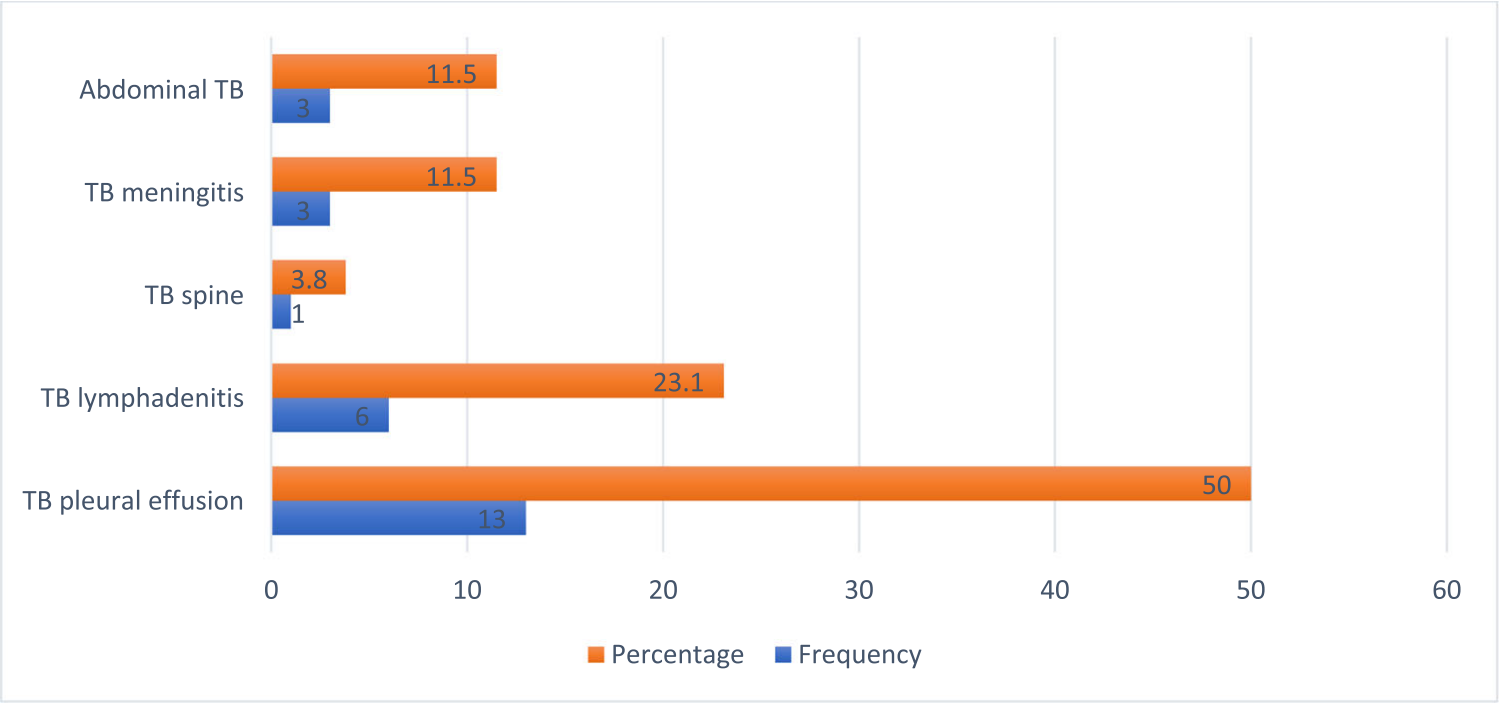
Type of extra‐pulmonary TB.

Among the 78 patients, 48 (61.5%) were cases of PTB followed by EPTB (*n* = 26; 33.3%) and disseminated TB (*n* = 4; 5.1%). Among cases of the EPTB (*n* = 26), the TB pleural effusion was the commonest presentation (*n* = 13; 50%) (Figure 2).

### 3.3 Modality of Diagnosis and Severity of TB

The majority of patients were clinically diagnosed (n=60; 77%), and only 18 (23%) were bacterologically confirmed TB. Severe TB was present in six (7.7%) patients (Table 1).

### 3.4 Prevalence and Clinical Presentation of Anti‐TB DILI

**Figure 3:**
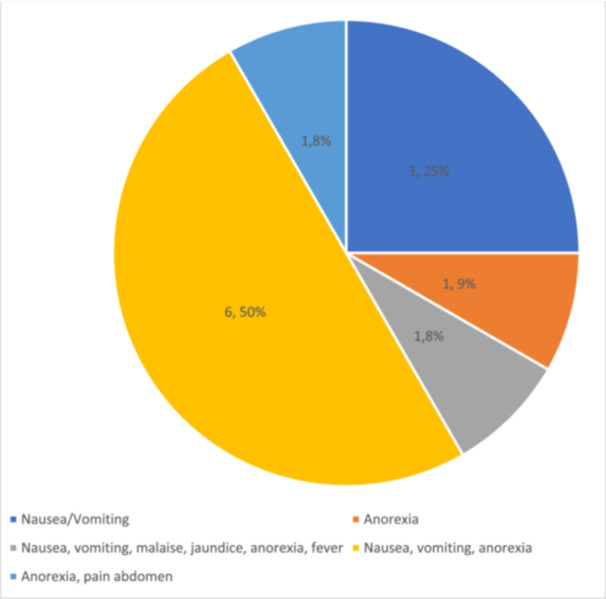
Clinical presentation of anti‐TB drug‐induced liver injury.

Out of the 78 patients who received ATT, 12 (15.4%) developed anti‐TB DILI during the intensive phase. Among the 12 patients of anti‐TB DILI, 6 (50%) presented with nausea, vomiting, and anorexia and 3 (25%) with nausea and vomiting (Figure 3).

### 3.5 Severity of Anti‐TB DILI

Among 12 patients of DILI, 6 patients (50%) had moderate DILI and only 1 patient (8.3%) had severe DILI (Table 2].

**Table 2:** Severity of anti‐TB drug‐induced liver injury.

Severity of anti‐TB DILI	Frequency	Percentage
Mild	5	41.7
Moderate	6	50
Severe	1	8.3

Abbreviations: DILI, drug‐induced liver injury; TB, tuberculosis.

### 3.6 Outcomes of Anti‐TB DILI

The majority of the patients with anti‐TB DILI improved after discontinuation of ATT (*n* = 11; 91.7%) and one patient died (8.3%).

### 3.7 Time of Development of Anti‐TB DILI

The median time of development of anti‐TB DILI after initiation of ATT was 11 (ranging from 7 to 60) days and the mean time of normalization of liver enzyme after discontinuation of ATT was 10.90 (SD = 6.45) days.

### 3.8 Recurrence Rate of Anti‐TB DILI

The recurrence rate of DILI after re‐initiation of ATT was 8.3% (one out of 12).

### 3.9 Risk Factors of Anti‐TB DILI

In our bivariate analysis, we identified hepatotoxic drugs, severe TB, low BMI, and low serum albumin as significant risk factors for anti‐TB DILI (*p* < 0.05). However, no statistically significant correlation was observed between anti‐TB DILI and age, gender, type of TB, modality of TB diagnosis, or co‐morbidities (*p* > 0.05) (Tables 3 and 4).

**Table 3:** Risk factors of anti‐TB drug‐induced liver injury.

Parameters	Patients with DILI (mean ± SD)	Patients without DILI (mean ± SD)	*p* value*
BMI (kg/m^2^)	19.90 ± 2.71	22.08 ± 3.47	0.025**
Serum albumin (g/dL)	3.38 ± 0.5	4.02 ± 0.99	0.002
Age (years)	54.91 ± 15.34	48.95 ± 19.11	0.25

Abbreviations: BMI, body mass index; DILI, drug‐induced liver injury; SD, standard deviation

* *p* value calculated using Mann–Whitney *U* test

** *p* value calculated using independent‐sample *t*‐test.

**Table 4:** Risk factors of anti‐TB drug induced liver injury.

Parameters	Number of patients with DILI (%)	Number of patients without DILI (%)	Total	*p* value*
Hepatotoxic drugs				
Yes	4	3	7	0.009
No	8	63	71	
Gender				
Male	6	39	45	0.752
Female	6	27	33	
Type of TB				
Pulmonary	7	41	48	0.35
Extrapulmonary	3	23	26	
Disseminated	2	2	4	
Modality of diagnosis of TB				
Bacteriological confirmed	2	16	18	0.722
Clinically diagnosed	10	50	60	
Comorbidity				
None	9	55	64	0.423
Diabetes mellitus	2	9	11	
Hypertension	1	2	3	
Severe TB				
Yes	3	3	6	0.044
No	9	63	72	

Abbreviations: DILI, drug‐induced liver injury; TB, tuberculosis.

* *p* value calculated using chi‐square test.

Upon conducting multivariable logistic regression analysis, we determined that hepatotoxic drugs (OR: 0.03, 95% CI: 0.003–0.42, *p* = 0.009), low BMI (OR: 1.42, 95% CI: 1.018–1.99, *p* = 0.039), and low serum albumin (OR: 3.31, 95% CI: 0.978–11.26, *p* = 0.054) independently predicted anti‐TB DILI (Table 5).

**Table 5:** Independent predictors of anti‐TB drug‐induced liver injury.

Parameters	OR	95% CI	*p* value
Gender	1.69	0.247–11.60	0.593
Comorbidity	2.18	0.006–741	0.739
Type of TB	1.84	0.048–71.78	0.743
Modality of diagnosis of TB	1.80	0.195–16.74	0.603
Severity of TB	0.076	0.002–2.44	0.146
Hepatotoxic drugs	0.03	0.003–0.42	0.009
Age	0.99	0.945–1.05	0.921
BMI	1.42	1.018–1.99	0.039
Serum albumin	3.31	0.978–11.26	0.054

Abbreviations: BMI, body mass index; CI, confidence interval; OR, odds ratio; TB, tuberculosis.

## 4 Discussion

The prevalence of TB remains elevated in developing nations, contributing significantly to morbidity and mortality. Short‐course ATT, comprising isoniazid, rifampicin, ethambutol, and pyrazinamide, has demonstrated remarkable efficacy in treating TB [3]. However, a primary drawback of ATT is DILI, which can result in treatment interruption, noncompliance, treatment failure, and the emergence of drug resistance.

In our investigation, we observed an incidence of anti‐TB DILI at 15.4% in Nepalese population. Comparable higher incidences of anti‐TB DILI were reported in studies conducted in Egypt (15%) [21], Ethiopia (13.8%) [22], and Pakistan (12.85%) [23]. Nonetheless, our findings contrast with population‐based study (*n* = 3900) conducted in China by Shang et al. (2.55%) [5]. Hospital‐based studies often involve participants with more complex and severe illnesses who undergo closer monitoring, thereby enhancing the likelihood of detecting hepatotoxicity. In our study, all patients underwent baseline LFTs before starting ATT. Follow‐up LFTs were conducted at the first and second weeks post‐initiation, with subsequent monitoring via phone contact at the fourth and eighth weeks to assess for hepatitis symptoms. Repeat LFTs were performed in symptomatic patients, who were encouraged to report any symptoms to their physician throughout the intensive phase of ATT. Ethnicity and genetic factors may also play significant roles. Several genetic variations in drug‐metabolizing enzymes have been associated with anti‐TB DILI, including slow acetylator status (*N*‐acetyl‐transferase 2), a glutathione *S*‐transferase M1 homozygote null genotype, and cytochrome P4502E1c1/c1 genotype [24]. Moreover, the prevalence of viral infections such as HIV and HCV can impact the incidence of DILI. Research suggests that hepatitis C patients have a fivefold higher relative risk of developing DILI, HIV‐positive patients have a fourfold risk, and patients coinfected with hepatitis C and HIV have a 14.4‐fold risk [25]. In our study, none of the patients tested positive for HIV, HBV or HCV infection.

In our study, half of the patients (50%) with anti‐TB DILI exhibited symptoms of nausea, vomiting, and anorexia (Figure 3). This aligns with findings from other studies conducted elsewhere, where these symptoms were frequently observed in cases of anti‐TB DILI [7, 22, 26]. Therefore, if a patient undergoing ATT presents with symptoms such as nausea, vomiting, or anorexia, it is advisable for the treating physician to consider the possibility of anti‐TB DILI and conduct appropriate investigations. Conversely, previous studies have indicated that asymptomatic transaminase elevations occur in 20% to 33.02% of patients undergoing standard anti‐TB regimens [5, 27, 28, 29]. However, in our study, none of the patients with anti‐TB DILI were asymptomatic. It is worth noting that patients with viral hepatitis may also manifest similar clinical features [30].

In our study, we identified that only 8.3% of patients experienced severe DILI (one out of 12), while the majority of patients had moderate DILI (50%). This contrasts with findings from other studies conducted elsewhere, where severe DILI was reported at rates ranging from 33.3% to 34.6% [22, 31]. This variation may be attributed to the relatively small sample size in our study and the rigorous and timely monitoring of our patients before the development of severe DILI.

In the current study, we found that the median time from the initiation of ATT to the development of anti‐TB DILI was 11 days (range: 7–60 days). This median onset time aligns closely with findings from hospital‐based studies conducted in Iran (14 days) [32] and Turkey (15 days) [33]. This similarity may be attributed to the more rigorous and precise monitoring of hospitalized patients. In contrast, population‐based studies reported longer median onset times, such as 38 days in a community‐based cohort study in Singapore and 52.5 days in a community‐based study in China [5, 34]. The timing of LFT monitoring may have influenced these findings, as in these studies, the majority of patients had their LFTs monitored over a period of 1–2 months.

We observed that the majority of our patients experiencing anti‐TB DILI showed improvement after discontinuation of ATT (*n* = 11; 91.7%), with only one patient (8.3%) died. Gezahegn et al.‘s study in North Ethiopia reported that 88.5% of patients improved after discontinuing anti‐TB treatment, with 11.5% experiencing mortality [22]. Similarly, a population‐based study conducted in China by Shang et al. revealed that 3.77% of patients died, while 96.23% showed improvement after discontinuing ATT [5]. Comparable findings were also reported in a study conducted in Egypt [21].

The median duration for liver enzyme normalization was 10.90 days (SD = 6.45). Comparable findings were noted in studies conducted in North Ethiopia (median time 14 days) and Egypt (within 2 weeks from cessation of therapy) [21, 22]. However, in our research, a recurrence of DILI was detected in 8.3% of cases (1 out of 12 patients), which contrasts with findings from studies in India (14%) [35]. This discrepancy may stem from the relatively limited sample size in our study. No recurrence was observed in studies conducted in North Ethiopia and New York [21, 25].

We determined that hepatotoxic drugs, low BMI, and low baseline serum albumin were independent predictors of anti‐TB DILI (Table 5). In accordance with findings from previous studies conducted in North Ethiopia, Iran, and India, the use of hepatotoxic drugs was notably linked to DILI [22, 36, 37]. The observation of a significant association between lower baseline serum albumin levels and the onset of DILI aligns with similar investigations conducted elsewhere [22, 38, 39, 40]. The study conducted by Fauzi et al. [40] in Malaysia revealed a notable correlation between lower BMI and DILI, a result that corresponds with the findings of our study.

Our study did not uncover a statistically significant correlation between the development of anti‐TB DILI and age or gender. Similarly, research conducted in North Ethiopia, Iran, and India also indicated no statistically significant association between anti‐TB DILI and age or gender [22, 32, 36]. In contrast, a separate study conducted in India identified a significant association between drug‐induced hepatitis and patients aged ≥ 60 years and female gender [39]. This variance could potentially be attributed to the older age of participants in the Indian study compared to ours, where the majority of participants belonged to younger age groups.

We found that the type of TB, its severity, and the diagnostic method used were not significantly linked to anti‐TB DILI. These results align with a study conducted in North Ethiopia by Gezahegn et al. [22]. However, our results contrast with findings from studies conducted in India and Ethiopia [32, 39, 41].

In studies carried out in Iran and Malaysia, HIV was identified as a risk factor for DILI [32, 40]. However, in our study, none of the patients tested positive for HIV. Consequently, we were unable to assess its statistical significance. In a Malaysian study, HBV infection was not linked to the development of anti‐TB DILI [42]. Conversely, in an Indian study, hepatitis B infection emerged as a significant risk factor for anti‐TB DILI [37]. However, in our study, none of the patients tested positive for HBV infection. Consequently, we were unable to assess the impact of HBV infection on anti‐TB DILI.

## 5 Strength and Limitation

The prospective nature of the study allowed for the systematic collection of data over a specified period, minimizing recall bias and providing more reliable information. Patients underwent comprehensive monitoring, including baseline LFTs, regular follow‐up assessments, and timely investigation of symptoms, enhancing the accuracy of detecting anti‐TB DILI and its outcomes. Patients were recruited from multiple departments within the hospital, ensuring a diverse sample representing different clinical presentations and severity levels of TB. The study adhered to established diagnostic criteria for anti‐TB DILI, providing consistency and comparability with other studies. Utilization of multivariable logistic regression allowed for the identification of independent risk factors for anti‐TB DILI, enhancing the understanding of its predictors in the Nepalese population.

The study's sample size was relatively small, which might limit the generalizability of the findings to the broader population. Conducting the study in a single tertiary care hospital may limit the generalizability of the results to other healthcare settings and populations within Nepal. The use of convenience sampling might introduce selection bias, as it relies on the availability and willingness of patients to participate. None of the patient tested positive for HBV and HCV infection, which could have provided additional insights into the association between viral infections and anti‐TB DILI. Since the patients were only followed up until the intensive phase of ATT, those who developed anti‐TB DILI during the continuation phase might not have been captured in the study. The study's follow‐up period of 60 days after anti‐TB DILI might not capture long‐term outcomes or recurrence rates beyond this timeframe. Overall, while the study provides valuable insights into the incidence, clinical features, outcomes, and associated factors of anti‐TB DILI in Nepal, these limitations should be considered when interpreting the results and designing future research.

## 6 Conclusion

In conclusion, this study sheds light on the incidence, clinical characteristics, outcomes, and associated factors of anti‐TB DILI among TB patients in Nepal. The findings underscore the significant burden of anti‐TB DILI in this population, with notable rates of incidence and moderate severity. Prompt recognition and management of anti‐TB DILI are crucial, as most patients showed improvement upon treatment discontinuation. However, the recurrence rate underscores the need for continued vigilance during treatment. Identified risk factors such as hepatotoxic drugs, low BMI, and low serum albumin levels can guide clinicians in identifying patients at higher risk for DILI, facilitating early intervention.

## 7 Recommendations

Based on our study's findings, we propose several recommendations to enhance the management of anti‐TB DILI and optimize treatment outcomes for TB patients. First, implementing regular liver function monitoring, particularly during the intensive phase of treatment, is crucial for early detection of anti‐TB DILI. Second, healthcare providers should be educated about anti‐TB DILI risk factors to ensure adherence to monitoring protocols. Additionally, alternative treatment regimens or closer monitoring should be considered for patients with identified risk factors. Conducting larger, multicenter studies is essential to validate these findings and explore additional factors influencing anti‐TB DILI. Standardized protocols for anti‐TB DILI management, including clear guidelines for treatment discontinuation and follow‐up, need to be developed and implemented. Lastly, public awareness about anti‐TB DILI symptoms should be enhanced to promote early reporting and intervention. By implementing these recommendations, healthcare systems can improve anti‐TB DILI management, reduce morbidity and mortality, and enhance treatment outcomes for TB patients, not only in Nepal but also globally.

## Author Contributions

**Ravi Ranjan Pradhan:** conceptualization, methodology, investigation, formal analysis, writing – original draft, writing – review and editing. **Ajay Kumar Yadav:** conceptualization, methodology, investigation, writing – original draft, writing – review and editing.

## Ethics Statement

Ethical clearance was obtained from institutional review committee (IRC) of Nepal health research council (NHRC) (Reg. No.: 321_2022).

## Consent

Prior written informed consent was taken from all the participants after explaining the nature and purpose of the study.

## Conflicts of Interest

The authors declare no conflicts of interest.

## Transparency Statement

The lead author Ravi Ranjan Pradhan affirms that this manuscript is an honest, accurate, and transparent account of the study being reported; that no important aspects of the study have been omitted; and that any discrepancies from the study as planned (and, if relevant, registered) have been explained.

## Data Availability

The data that support the findings of this study are openly available in Figshare at https://doi.org/10.6084/m9.figshare.25975345.
